# Motor Neuron Synapse and Axon Defects in a *C. elegans* Alpha-Tubulin Mutant

**DOI:** 10.1371/journal.pone.0009655

**Published:** 2010-03-11

**Authors:** Renee Baran, Liliana Castelblanco, Garland Tang, Ian Shapiro, Alexandr Goncharov, Yishi Jin

**Affiliations:** 1 Biology Department, Occidental College, Los Angeles, California, United States of America; 2 Section of Neurobiology, Division of Biological Sciences, University of California San Diego, San Diego, California, United States of America; 3 Howard Hughes Medical Institute, University of California San Diego, San Diego, California, United States of America; Columbia University, United States of America

## Abstract

Regulation of microtubule dynamics underlies many fundamental cellular mechanisms including cell division, cell motility, and transport. In neurons, microtubules play key roles in cell migration, axon outgrowth, control of axon and synapse growth, and the regulated transport of vesicles and structural components of synapses. Loss of synapse and axon integrity and disruption of axon transport characterize many neurodegenerative diseases. Recently, mutations that specifically alter the assembly or stability of microtubules have been found to directly cause neurodevelopmental defects or neurodegeneration in vertebrates. We report here the characterization of a missense mutation in the C-terminal domain of *C. elegans* alpha-tubulin, *tba-1(ju89)*, that disrupts motor neuron synapse and axon development. Mutant *ju89* animals exhibit reduction in the number and size of neuromuscular synapses, altered locomotion, and defects in axon extension. Although null mutations of *tba-1* show a nearly wild-type pattern, similar axon outgrowth defects were observed in animals lacking the beta-tubulin TBB-2. Genetic analysis reveals that *tba-1(ju89)* affects synapse development independent of its role in axon outgrowth. *tba-1(ju89)* is an altered function allele that most likely perturbs interactions between TBA-1 and specific microtubule-associated proteins that control microtubule dynamics and transport of components needed for synapse and axon growth.

## Introduction

Microtubules play multiple essential roles in cells including forming the mitotic spindle, transporting vesicles and organelles, providing structural support as part of the cytoskeleton, and serving as platforms for the assembly of signaling complexes. In neurons, the transport functions of microtubules are especially crucial for establishing neuron morphology and maintaining synapses distant from the cell body. Evidence from genetic model systems has revealed that microtubules also play key roles in synapse growth and plasticity [1]–[4], although these mechanisms are not fully defined.

Microtubules are composed of alpha- and beta subunits that are assembled into heterodimers with the help of cofactors and are then incorporated into microtubule polymers [5]–[6]. Like actin, microtubules are dynamic polymers, and their assembly and disassembly is highly regulated. Organization into complex microtubule arrays and integration with the actin cytoskeleton adds additional layers of complexity that are still poorly understood. Misregulation of microtubules, loss of synapses and breakdown of transport mechanisms have long been recognized as common denominators in the pathology of neurodegenerative diseases. The presence of hyper-phosphorylated tau in the neurofibrillary tangles of Alzheimer's patients is one of the most intensely studied examples, but an increasing number of studies have established that perturbing any of the major mechanisms that regulate microtubule dynamics and transport can impact neural development or contribute to neuronal disease. Examples include mutations in tubulin folding co-factor E [7], the dynactin complex [8]–[9] and the microtubule severing protein, spastin [10], which have all been implicated in motor neuron degeneration.

Early studies of microtubule dynamics and function focused on the beta-tubulin subunit because of its role in hydrolyzing GTP during microtubule polymerization and position on the exposed, plus-ends of microtubules [5]. Subsequent research has uncovered important roles for the alpha-tubulin subunit in synaptic plasticity and neuronal disease. Alpha-tubulin mRNA is enriched at *Aplysia* synapses and translated locally in response to serotonin signaling [11]. In vertebrates, alpha-tubulin is a target of the E3 ligase and Familial Parkinson's Disease protein Parkin [12]–[13]. Perturbations of plus-end microtubule binding proteins of the ebp l family, which can bind the alpha-tubulin subunit [14], have also been linked to motor neuron degeneration [15]. Recently, mutations in human and mouse TUBA1A(TUBA3) alpha-tubulins were identified that block cell migration in the mammalian cortex, possibly by altering interactions between the alpha-tubulin subunit and the microtubule-nucleating and stabilizing protein doublecortin [16].

We describe here the isolation and characterization of a novel gain-of-function allele of the *C. elegans* alpha-tubulin, *tba-1. tba-1(ju89)* mutants exhibit a reduction in the number and size of GABAergic and cholinergic motor neuron synapses, altered locomotion and axon extension defects. We show similar axon outgrowth defects also occur in mutants in which the *C. elegans* beta-tubulin TBB-2 is deleted. Genetic analysis suggests that many of the axon phenotypes observed in *ju89* mutants are likely mediated by altered or reduced function of microtubules composed of TBA-1 and TBB-2 subunits. We propose that *ju89* alters synapse and axon growth by interfering with specific microtubule-associated proteins that control microtubule dynamics and stability and regulate transport of structural components of axons and the presynaptic active zone.

## Results

### Isolation of *ju89* and characterization of synapse and axon defects

**Figure 1:**
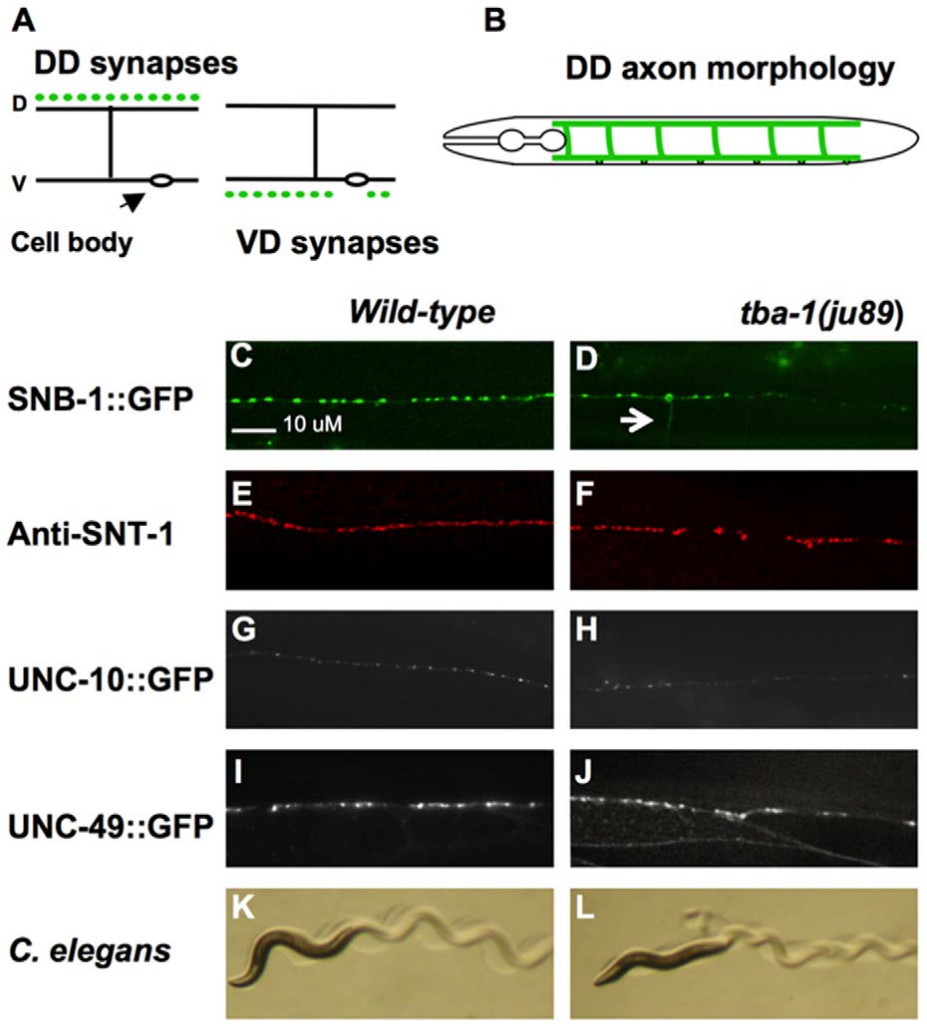
GABAergic Motor Neuron Synapses are Defective in *tba-1(ju89*) Mutants. (A) Diagram of *en passant* neuromuscular synapses in *C. elegans*. Dots represent synapses; dorsal D neurons (DDs) form synapses onto dorsal body-wall muscles, and ventral D neurons (VDs) synapse with ventral muscles. Presynaptic termini of the inhibitory GABAergic type D motor neurons are visualized with the synaptic vesicle marker P*_unc-25_*SNB-1::GFP. Each GFP puncta visible by fluorescence microscopy corresponds to the cumulative signal from all GFP-tagged vesicles at an individual synapse. (B) Morphology of the six DD motor neurons. Cell bodies are located in the ventral nerve cord. Each D neuron extends a ventral process, branches to form a commissure, and bifurcates at the dorsal nerve cord to form a dorsal process along the dorsal nerve cord. At the distal tip of each process, gap junctions are formed with adjacent DD neurons [17]. Expression pattern of SNB-1::GFP in wild-type (C) and mutant (D) dorsal nerve cord. Irregular size puncta and reduced numbers of puncta are evident in *ju89* mutants. An arrow designates the position of a DD commissure. (E) SNT-1 (synaptotagmin) expression in the dorsal nerve cord of wild-type and (F) *ju89* mutant animals. UNC-10::GFP expression along the dorsal nerve cord of (G) wild-type and (H) *ju89* worms. (I) Expression of the GABA-B receptor subunit, UNC-49::GFP, along dorsal muscles of wild-type animals and (J) *ju89* mutant worms. Gaps in UNC-49::GFP expression demonstrate the lack of post-synaptic structures in these regions. Wild-type *C. elegans* (K) have a larger body size than *tba-1(ju89)* (L). *C. elegans* move in a wavelike pattern. *ju89* mutants are uncoordinated, and the amplitude of the wave pattern is severely reduced compared to wild-type animals.

The GABAergic D motor neurons function as cross-inhibitors of body wall muscle contractions in *C. elegans* and share a similar unipolar morphology (Figure 1A). Each D neuron extends an anterior process along the ventral cord, branches to form a commissure and then branches again at the dorsal side and extends in both directions along the dorsal nerve cord. Reconstruction of adult synaptic connectivity from electron micrographs show the six dorsal D neurons (DDs) receive synaptic input from cholinergic motor neurons on the ventral side and form chemical synapses en passant with dorsal muscles. The 13 ventral D neurons (VDs) receive their input from dorsal cholinergic neurons and synapse onto the ventral muscles [17].

Synapses can be visualized in the 19 D type GABAergic motor neurons by driving expression of the synaptic vesicle marker synaptobrevin::GFP (SNB-1::GFP) with the promoter of the *C. elegans* GAD (glutamic acid decarboxylase) gene, *unc-25* [18], [19]. The P*_unc-25_*SNB-1::GFP marker (*juIs1)* appears as puncta of mostly uniform size and spacing along the ventral and dorsal nerve cords of wild-type animals (Figure 1C), corresponding to the GABAergic synapses defined by EM analysis [17]. *ju89* was isolated from a genetic screen for mutations that alter the P*_unc-25_*SNB-1::GFP expression pattern [20]. This approach successfully identified synapse defective (syd) genes that function in active zone formation and synapse morphology [21]–[22], neuronal polarity [20], [23] and vesicle transport [24].

*ju89* worms exhibited multiple defects in the SNB-1::GFP pattern along both the ventral and dorsal nerve cords that suggested a failure to properly form or maintain synapses. The number of SNB-1::GFP puncta was greatly reduced, and the SNB-1::GFP puncta exhibited greater variability in size and spacing than synaptic GFP-puncta of wild-type animals (Figure 1C & D). The number of DD puncta along the dorsal nerve cord was reduced by an average of 42% in young adult *ju89* worms cultivated at 20°C, and the number of VD puncta in the ventral nerve cord was reduced by 34% (Table 1). Loss of SNB-1::GFP appeared most severe at the distal tips of the dorsal D neurons, although irregular size SNB-1::GFP puncta occurred all along the dorsal cord. The majority of abnormal SNB-1 puncta were smaller than wild-type puncta, but unusually large GFP puncta were sometimes also observed along the dorsal cord. Weak diffuse GFP was also observed along commissures (Figure 1D), a phenotype that is associated with mislocalization of synaptic vesicles to these regions and that has been observed in other syd mutants, such as syd*-2*/liprin [21]. All *ju89* adult worms showed uncoordinated (unc) movement, consistent with defects in synaptic transmission in the motor circuit (Figure 1L).

**Table 1:** SNB-1::GFP Expression by D Motor Neurons.

Genotype	Ventral Nerve Cord (VDs)	(n)	Dorsal Nerve Cord (DDs)	(n)
+/+	189±14	30	166±10	30
*tba-1/(ju89)*	125±22*	30	94±14*	30
*tba-1(ju89)/+*	169±15*	30	148±8*	30
*qDf9/ju89*	148±15*	10	122±12*	10
*qDf9/+*	187±6	15	168±12	15
*tba-1(ok1135)*	192±6	30	167±7	30
*tba-1(ok1135)/+*	180±9	30	164±9	30
*tba-1(ok1135)/ju89*	155±7*	15	130±8*	15
*+:* Ex*tba-1(R414)*	nd		107±3*	25
*+; Extba-1*	nd		166±7	25
*tbb-2(gk130)*	153±10*	30	131±14*	30
*tba-1/(ju89);tbb-2(gk130)*	148±16*	30	117±13*	30
*tbb-1(gk207)*	173±8*	30	158±7*	30
*tba-1(ju89); tbb-1(gk207)*	76±12**	30	65±14**	30

Genetic analysis of tubulin mutant synaptic defects based on expression pattern of the synaptic vesicle marker Synaptobrevin::GFP (SNB-1::GFP). Young adult worms were scored for the number of SNB-1::GFP puncta in the ventral and dorsal nerve cords. Animals carrying the *ju89* mutant chromosome in trans to a wild-type (*+/ju89)* or deficiency (*Df9/ju89)* chromosome or the *tba-1* deletion allele (*ok1135/ju89*) exhibit partial defects. Deficiency/+ and *ok1135/+* heterozygotes appear wild-type. Animals that express an extrachromosomal array containing *tba-1* amplified from *ju89* animals, *Extba-1(R414)* resemble *ju89* mutants. Animals homozygous for the null *tbb-2* allele *(gk207)* have a similar decrease in total SNB-1::GFP as single mutants and in combination with *tba-1(ju89)*. Mean ± sd.

*p<.001, two-tailed T test.

**Synapse loss is strongly enhanced in *tba-1(ju89); tbb-1 (gk207)* animals due to loss of axons in the dorsal and ventral nerve cord.

To determine whether the SNB-1::GFP localization represented defects in synapse and axon differentiation and were not due primarily to a failure in synaptic vesicle transport or docking at otherwise normal presynaptic specializations, we examined the distribution of a post-synaptic GFP-tagged-GABA receptor subunit (UNC-49::GFP) in the body wall muscles [25]. UNC-49::GFP is expressed by both ventral and dorsal body wall muscles and appears as diffuse puncta adjacent to the ventral and dorsal nerve cords (Figure 1I & J). Large gaps in UNC-49::GFP expression were evident along both the dorsal and ventral muscles of adult *ju89* mutants, indicating that postsynaptic structures were not present in these regions. In some *ju89* mutant animals the GFP-tagged receptor appeared diffused throughout the muscle tissue, consistent with the failure of the receptor to completely localize to synapses.

To assess if presynaptic specializations were still present in these regions of the motor neuron axons, we examined the expression of a GFP-tagged active zone protein, UNC-10(RIM). In the presynaptic matrix, UNC-10 binds the scaffolding protein ELKS/ERC and interacts with the RAB-3 GTPase to recruit synaptic vesicles to the active zone [26]. Because the *C. elegans* cholinergic motor neurons run closely parallel to the GABAergic D motor neurons in the ventral and dorsal nerve cords, the synapses of the two classes of motor neurons cannot be distinguished from each other by immunohistochemistry and light microscopy. To view UNC-10 expressed by only the GABAergic neurons we crossed P*_unc-25_*UNC-10::GFP (*hpIs61*) [27] into *ju89* mutants, and compared the localization of UNC-10::GFP puncta in *ju89* mutants to wild-type worms. Puncta for UNC-10::GFP appeared as an orderly row along the dorsal and ventral nerve cords in wild-type animals (Figure 1G). The pattern of UNC-10::GFP puncta of *ju89* mutants (Figure 1H) was similar to the mutant SNB-1 pattern: UNC-10::GFP puncta were frequently smaller than wild-type and irregularly spaced, and diffuse UNC-10::GFP was visible in the commissures of adult *ju89* animals. Again, the most distal tips of the DD axons appeared to be thinner and lacked full size UNC-10::GFP puncta. Thus, the presynaptic regions of GABAergic synapses were defective in both synaptic vesicles and the machinery required to recruit them.

To determine if all synapses were generally defective in *ju89* mutants, we also examined mutant worms for the endogenous expression of a second synaptic vesicle marker synaptotagmin (SNT-1) (Figure 1E & F) and UNC-10/RIM. SNT-1 and UNC-10 antibodies detect all chemical synapses in the *C. elegans* nervous system. In the most severe cases, anti-SNT-1 Ab revealed occasional gaps and irregular size or position of SNT-1 puncta in *ju89* mutants (Figure 1F), but expression of SNT-1 in mutant dorsal nerve cords appeared similar to wild-type SNT-1 (Figure 1E) expression in most animals, indicating that cholinergic motor neuron synapses are not globally disrupted in the mutants. Similar results were obtained using antibodies against UNC-10/RIM (data not shown).

**Figure 2:**
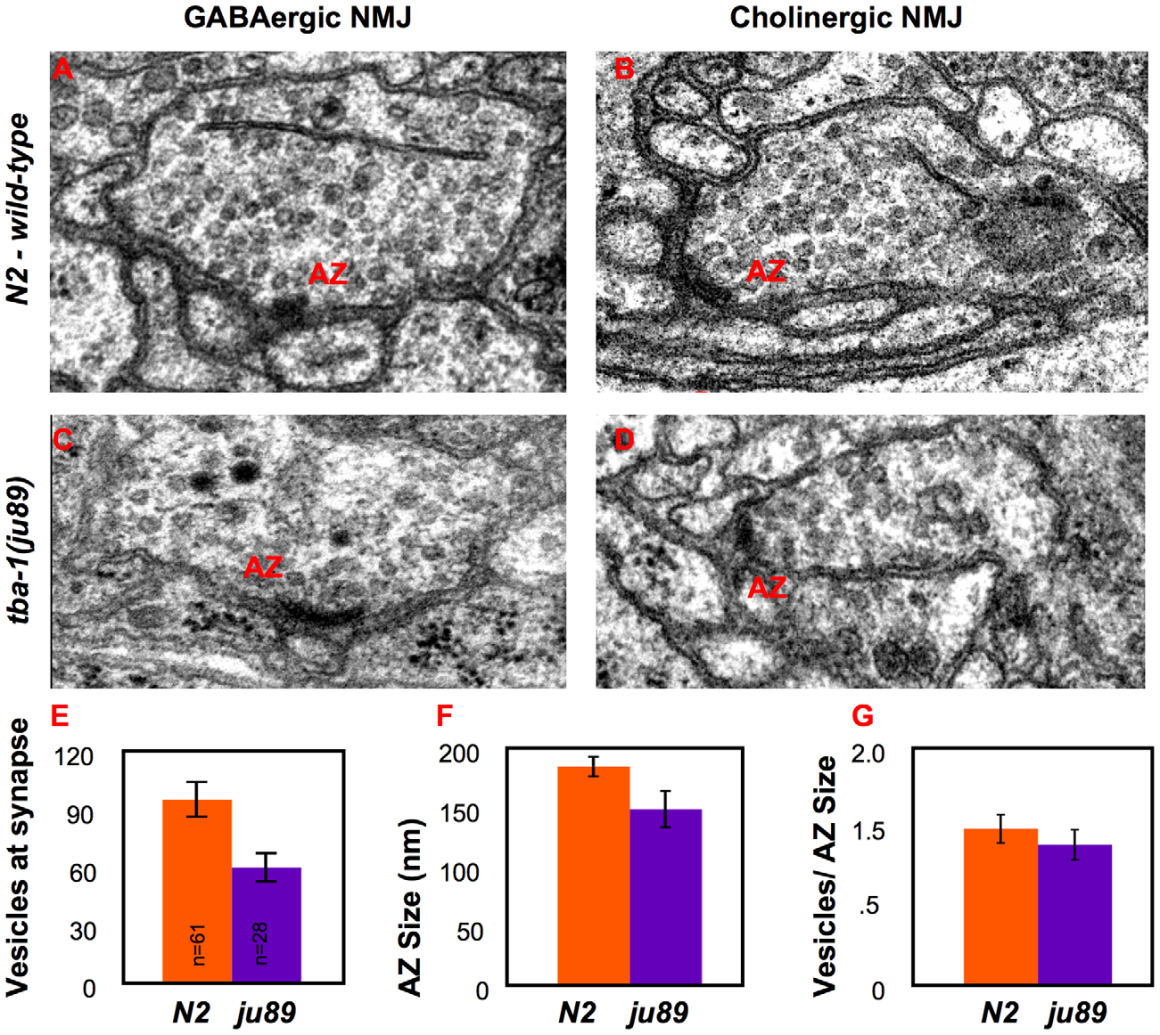
Ultrastructure of *tba-1(ju89)* Motor Neuron Synapses. The ultrastructure of one adult wild-type and two *ju89* adult mutant worm were examined by EM (see Methods). 1500 continuous sections of 50 nm each were collected from 1 wild-type adult animal and 400 sections from each of two *ju89* adult mutant worms. Sections were collected in the anterior of each animal between the nerve ring and the vulva. Inhibitory GABAergic motor neurons form synapses directly onto dorsal and ventral muscles; excitatory cholinergic motor neurons form dyadic synapses with muscles and a GABAergic motor neuron dendrite. Representative GABAergic motor neuron synapses with body wall muscle arms in (A) wild-type and (C) mutant *tba-1(ju89)* animals, and cholinergic motor neuron synapses in (B) wild-type and (D) *ju89* worms. Fewer synaptic vesicles are visible in both types of synapses in the mutants. (E) The number of synaptic vesicles per active zone (p<.05), (F) length of the active zone in nm (p<.05), and (G) number of synaptic vesicles/active zone length for the cholinergic motor neuron synapses in one animal are shown. Cholinergic synapse size is reduced in the *ju89* mutant. Error bars: S.E.M.

Previous studies have shown that significant changes in expression of SNB-1::GFP often reflect underlying morphological changes at synapses [21]–[22]. Our GFP transgene expression data indicated that synapse formation and size could be altered in *ju89* mutants, and that GABAergic neurons were most severely affected. To determine if the small SNB-1::GFP puncta in *ju89* mutants correlated with morphological defects at synapses, we analyzed *ju89* animals by electron microscopy. Compared to wild-type synapses (Figure 2A & B), a reduction in the number of synaptic vesicles is apparent in electron micrographs of both GABAergic (Figure 2C) and cholinergic synapses of the mutants (Figure 2D). An average of 20–30% fewer vesicles/synapse and an equivalent decrease in active zone size was measured in the cholinergic synapses of one animal, (Figure 2E & F), but the proportion of vesicles/active zone size was similar in wild-type and mutant animals (Figure 2G). The loss of GABAergic synapses was severe in the regions examined: the number of dorsal synapses in a section of the dorsal cord in one animal was reduced by 2/3 compared to a similar sized region in the wild-type control (2 synapses in *ju89* vs. 6 synapses in the wild-type animal). When we examined the total number of GABAergic and cholinergic motor neuron axons present in the dorsal and ventral nerve cords, the number of axons in the ventral nerve cord was reduced by 8% compared to wild-type worms (49 versus 53 axons), and the number of axons in the dorsal nerve cord was reduced by 16% (9 versus 12 axons). This observation indicated that some of the mutant motor neuron axons failed to reach or extend normally along the dorsal nerve cord.

**Figure 3:**
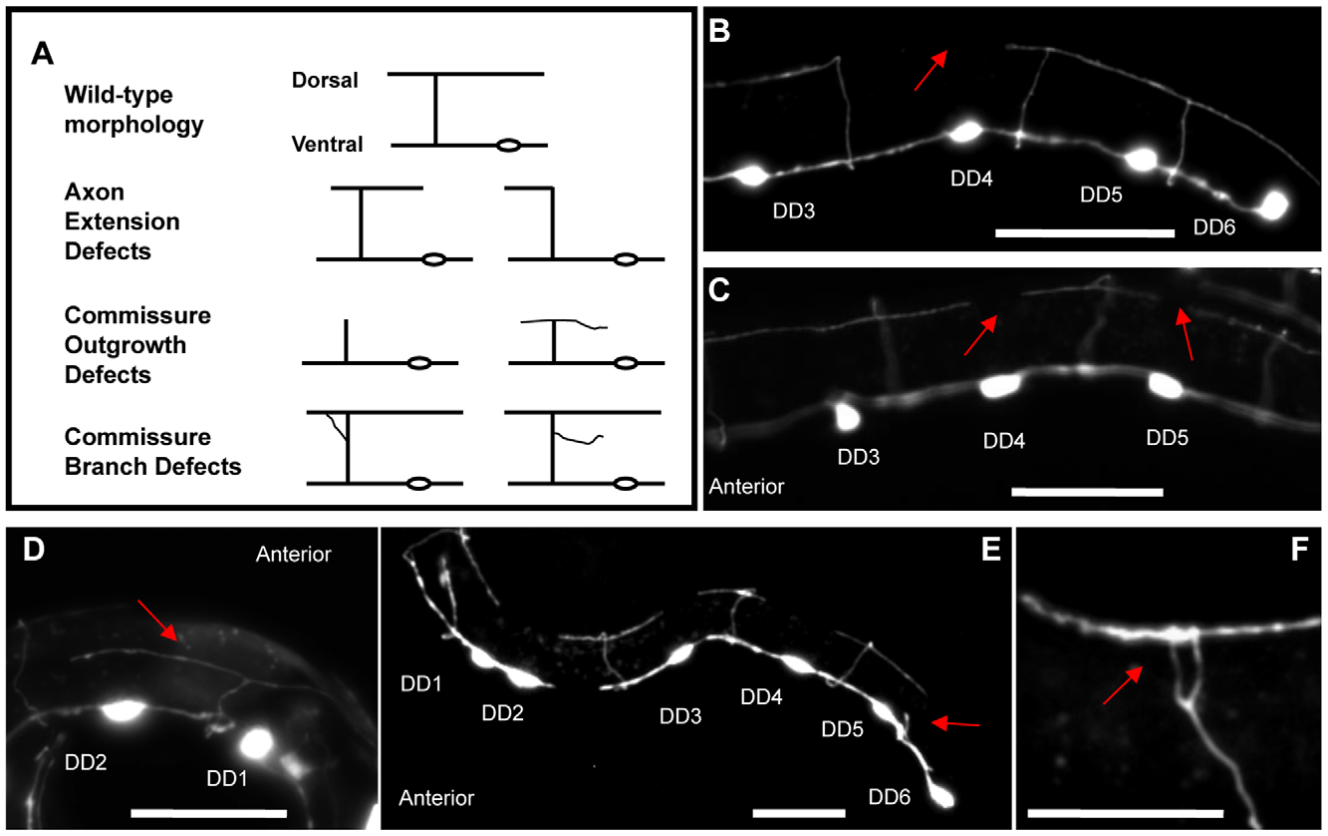
Axon Outgrowth Defects in Tubulin Mutants. (A) Diagrams of defects in axon extension and commissure outgrowth observed in newly hatched mutant worms (see Table 2). The morphology of the DD neurons in wild-type and mutant worms was visualized with the cytoplasmic axon markers P*_unc-25_*GFP or P*_flp-13_*GFP. Examples of axon defects in tubulin mutants: (B), (C) short regions (arrows) where axons fail to extend to their full length along the dorsal nerve cord of *tba-1(ju89)* worms; (D) a commissure branches prematurely and fails to reach the dorsal nerve cord in a *tbb-1(gk207)* mutant animal; (E) axon outgrowth or extension defects in all six DD neurons of a newly hatched L1 larvae. The DD6 commissure stalls prematurely (arrow), and the DD2 cell body is also displaced anterior. (F) abnormal commissure branch (arrow) in a *tba-1(ju89)* mutant. Scale bar 20 µM.

**Figure 4:**
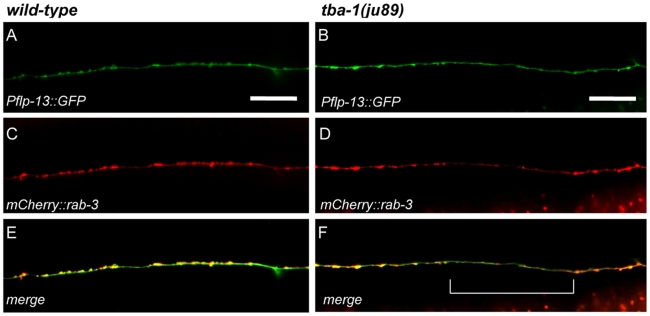
Synapse Loss in DD Axons of *tba-1(ju89)* Animals. The DD neuron axon marker P*_flp-13_*::GFP was coexpressed with a presynaptic marker P*_unc-25_* mCherry::RAB-3 in wild-type (A, C, E) and mutant (B, D, F) *tba-1(ju89)* animals (see Methods). Axon extension was similar in both the wild-type (A) and mutant (B) axons depicted. mCherry::RAB-3 puncta were reduced or missing in the *tba-1(ju89)* mutant axon (D, F) compared to the wild-type worm (C, E). The bracket in (F) delineates a region in which mCherry::RAB-3 puncta are missing or greatly reduced in size in the mutant. Scale bar 20 µM.

To determine if the decreased number of GABAergic synapses in *ju89* mutants was due solely to defects in initial axon outgrowth and extension along the nerve cords, we examined the morphology of the dorsal D (DD) motor neurons in more detail. The six DD motor neurons are born and extend axons during embryogenesis, and can be visualized in newly hatched L1 larvae using P*_unc-25_GFP*. The cell body, axons and dendrites of DD neurons can be visualized in adult animals expressing P*_flp-13_GFP* [28]–[29]. This GFP marker is expressed by the six dorsal D neurons, but not by any of the cholinergic body wall motor neurons or the ventral D (VD) GABAergic neurons that are born at the end of the L1 stage. The number of DD axons in newly hatched L1 mutant worms that failed to reach the dorsal nerve cord increased by only 2% compared to wild-type controls, but 20% of mutant DD L1 axons (approximately 1 axon per animal) failed to extend their full distance along the dorsal cord (Table 2; Figure 3). The range of DD outgrowth defects observed in *ju89* mutants is shown in Figure 3 (A–D). Sixteen% of *ju89* L1s exhibited no outgrowth defects, while one or two small dorsal gaps were documented in an additional 62% of the L1 mutants sampled (Figure 3B & C). When the length of the gaps in each L1 animal was measured and compared to the total length of the DD axons along the dorsal nerve cord, DD axon extension along the dorsal nerve cord was reduced an average of 11.2% in *tba-1(ju89)* mutants at the L1 stage compared to 1.1% in the wild-type controls (Table 2). To analyze the extent of synapse loss due to missing axons in adult worms, we examined worms that expressed SNB-1::GFP exclusively in the DD neurons (P*_flp-13_*SNB-1::GFP) and also constructed wild-type and mutant strains that expressed both an axon marker for the DD neurons (P*_flp-13_*GFP) and the presynaptic reporter gene P*_unc-25_*mCherry::rab-3 (Figure 4). Axonal regions containing reduced numbers of mCherry::rab-3 were present in all *ju89* worms three days post-hatching (Figure 4D–F), a phenotype not observed in the wild-type controls (Figure 4A–C). DD axon extension along the dorsal nerve cord was reduced an average of 17% (s.e.m. ±6, n = 30) in young adult *ju89* mutants. These results were consistent with the decreased number of dorsal GABAergic axons of *ju89* adults observed by ultrastructure analysis.

**Table 2:** DD Neuron Outgrowth and Stability.

	Animals affected	Axons affected	% Dorsal cord missing	Commissure outgrowth defects	Commissure branching defects
**Axon outgrowth L1 larvae**					
*wild-type*	8/33 (24%)	10/198 (5%)	1.1	0/198 (0%)	0/198 (0%)
*tba-1(ju89)*	44/52 (85%)*	62/311 (20%)*	11.2	7/302 (2%)	0/312 (0%)
*tbb-2(gk130)*	54/57 (95%)*	59/342 (17%)*	8.2	2/342 (<1%)	0/342 (0(%)
*tba-1(ju89);tbb-2(gk130)*	47/54 (87%)*	55/324 (17%)*	7.6	3/324 (<1%),	1/324 (<1%)
*tbb-1(gk207)*	27/50 (54%)*	34/250 (13.6%)*	4.5	3/250 (<1%)	1/250(<1%);
*tba-1(ju89);tbb-1(gk207)*	43/50 (86%)*	79/250 (32%)*	20.5	15/250 (6%)*	1/250 (<1%)
**Axon stability Young Adult**					
*wild-type*	6/100 (6%)	10/500 (2%)	nd	2/500 (<1%)	2/500 (<1%)
*tba-1(ju89)*	11/45 (24%)*	18/225 (8%)*	nd	6/225 (3%)**	15/225 (7%)*

The axon morphology of the six dorsal D (DD) motor neurons was visualized with P*_flp-13_*GFP *(juIs145)* and P*_unc-25_*GFP (*juIs76)* transgenes in L1 larvae and with P*_flp-13_*GFP in young adults (see Methods). All 6 DD axons were evaluated in animals expressing *juIs76*, and DD1-DD5 axons were examined in worms that expressed the *juIs145* reporter. Animals were scored for gaps along the dorsal nerve cord, commissures that stopped short of the dorsal nerve cord, and abnormal branching morphology where commissures join the dorsal nerve cord (Figure 3). *tbb-2(gk130)* mutants and *tba-1(ju89);tbb-2(gk130)* animals exhibit similar DD axon defects, whereas axon defects are increased in *tba-1(ju89);tbb-1(gk207)* double mutants.

*Two-tailed Z-test, p<.005.

**p<.05.

Lastly, we asked if the severity of synapse and axon loss increases as mutant animals grow to adulthood. Between the L1 larval stage and the adult stage, *C. elegans* increases in size by fourfold. During this period the DD motor neurons add new membrane and grow in size but do not extend growth cones and undergo new axon outgrowth. The dorsal synapses of the DD neurons first form at the L1/L2 larval transition, after the initial outgrowth of these neurons [17], [20]; addition of new synapses and membrane to enlarge the neurons occurs during subsequent larval stages [27]. When we compared young adult wild-type and *ju89* mutant worms, we observed an increase in the number of DD axons with gaps in the dorsal nerve cord, and 7% of the DD axons in adults exhibited defects in branching where the commissures meet the dorsal cord that were not observed in the same animals at the L1 stage (Table 2, Figure 3F).

The loss of GABAergic synapses in *ju89* young adult worms thus appears more severe than can be accounted for solely by defects in initial axon outgrowth, suggesting that *ju89* may disrupt synapse formation and function independently of an earlier role in neuron outgrowth or guidance. The increases in DD branching and gaps in the dorsal cord observed in adult *ju89* animals may represent failure of the axon to grow (inserting new membrane and synaptic components) as mutant animals mature to their adult size. Alternatively, the DD motor neuron morphology may be unstable in adults due to defective differentiation of the axon during the earlier outgrowth phase.

### *ju89* alters a conserved residue between helix 11 and helix 12 in alpha-tubulin TBA-1

**Figure 5:**
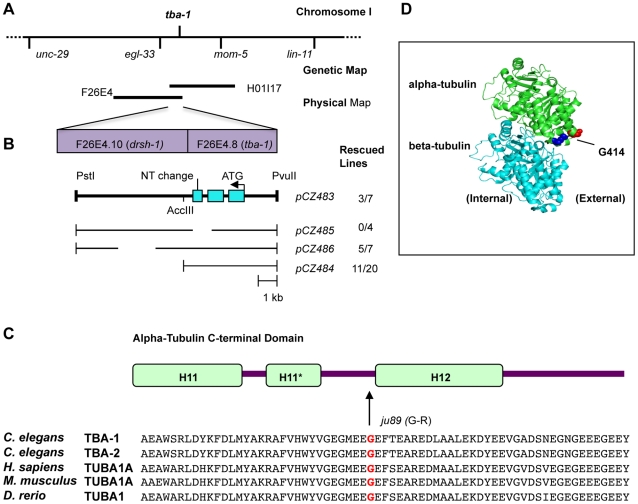
*ju89* is a Novel Allele of *C. elegans* Alpha-Tubulin *tba-1*. (A) location of *ju89* on *C. elegans* chromosome I based on genetic map data, (B) Transformation rescue of *ju89* by F24E4.8 (*tba-1)*. The minimal rescuing activity for *ju89* was narrowed to an overlapping region of cosmid F26E4 and fosmid HO1I17 containing the *tba-1-drsh-1* operon. *pCZ485*, a 4.5 kb subclone containing upstream sequence and only the *tba-1* coding region (F26E4.8) is sufficient to rescue the SNB-1:GFP and locomotion defects of *ju89* mutants. (C) *ju89* is a missense mutation that converts a conserved glycine to arginine in the H11–H12 loop of the TBA-1 C-terminus. C*-*terminal domain structure based on Lowe et al., 2001. Sequences used for the alignment are *C. elegans* TBA-1 (CAB03001), TBA-2 (CAB16856), human TUBA3/TUBA1A (NP006000), *Mus musculus* TUBA1A (AAH83344), and *Danio rerio* (NP919369). (D) Crystal structure of alpha-beta tubulin dimer generated by Polyview based on Nogales et al, 1998 and Lowe et al., 2001 (PDB#1JFF). Red highlights the residue altered in *ju89* mutants (G414 in TBA-1 and G416 in TUBA1A) located at the beginning of helix 12. Blue highlights the R402 residue mutated in human lissencephaly (R400 in TBA-1) in the H11–H12 loop adjacent to H11.

We mapped the *ju89* mutation between *egl-33* and *lin-11* on *C. elegans* chromosome I by standard two- and three-factor mapping (Figure 5A) (see Experimental Procedures). Both the SNB-1::GFP defects and uncoordinated movement of *ju89* mutants were rescued with fosmid H01I17 and cosmid F26E4 (Figure 5B). We subsequently narrowed the minimal rescuing region to a 10.5 kb genomic clone that contained the two-gene operon encoding *tba-1*(F26E4.10), and *drsh-*1 (F26E4.8). Genomic subclones that contained only *tba-1*, the first gene of the operon, were sufficient to rescue the *ju89* mutant defects. Deletions in *tba-1*, but not *drsh-1*, abolished the rescuing activity (Figure 5B).

**Figure 6:**
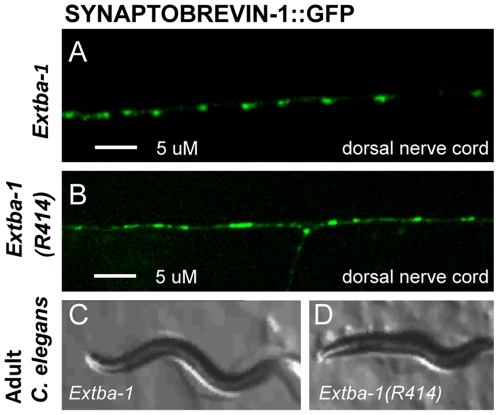
Expression of Mutant *tba-1(ju89)* from Extrachromosomal Arrays in Wild-Type *C. elegans* Phenocopies *tba-1 (ju89)* Defects. PCR products corresponding to the *tba-1* 4.5 kb rescuing region of *tba-1* were amplified from wild-type (N2) or *ju89* mutant animals and injected into wild-type *juIs1* hermaphrodites to generate transgenic lines (see Methods). 14/27 lines expressing the mutant *tba-1* gene, *Extba-1(R414)* exhibited uncoordinated movement defects and altered SNB-1::GFP patterns similar to *ju89* homozygotes. All seven lines expressing wild-type *tba-1* (*Extba-1*) were wild-type. Expression of the *juIs1* SNB-1::GFP transgene in the dorsal nerve cord of wild-type *N2* worms expressing (A) *Extba-1*; or (B) *Extba-1(R414)*. The body size and locomotion of transgenic animals expressing wild-type *tba-1* arrays (C) was the same as wild-type worms, whereas *N2* worms expressing arrays of *tba-1* amplified from the *ju89* mutant (D) exhibited the smaller body size and uncoordinated movement characteristic of *ju89* mutants. Scale bar 5 µM.

*tba-1* encodes one of nine predicted alpha-tubulins in *C. elegans* [30]. TBA-1 and its close paralogue, TBA-2, are both required maternally and are expressed by all cells of the early embryo [31]. TBA-1 continues to be expressed by neurons in adults [32]. Microtubules are composed of heterodimers of alpha-tubulin and beta-tubulin that are preassembled before addition to the growing plus ends of microtubule polymers. All alpha-tubulin isoforms are highly homologous in sequence, with the greatest variation occurring in the C terminal domain that binds motor proteins and other microtubule-associated proteins. The crystal structure for a human alpha-tubulin is defined [33]. Sequence analysis of *tba-1* in *ju89* mutant worms identified a point mutation that changes a conserved glycine to arginine (G414R) (Figure 5C & D). Based on the crystal structure of the human tubulin dimer [33]–[34], the *ju89* mutation is located in the C-terminal domain of alpha-tubulin in a loop near the final alpha-helix, H12 (Figure 5C & D). The mutation is predicted to alter the external architecture of the microtubule polymer and can thereby influence the binding of motor proteins and other microtubule-associated proteins.

### The *tba-1(ju89)* allele has altered gene function

Dominant mutations in a closely related human alpha-tubulin, TUBA1A (TUBA-3), are associated with defects in neuron migration and axon tract formation and are proposed to be due to haploinsufficiency of TUBA1A/TUBA3 [16]. Because the *ju89* mutation may cause a structural change in TBA-1, we asked if it behaved as a dominant, altered function mutation or exhibited haploinsufficiency. Wild*-type C. elegans* move in a sinusoidal wave pattern, whereas *ju89* homozygotes were uncoordinated, and the amplitude of the wave pattern was greatly reduced when animals move forward (Figure 1K & L). This visible locomotion phenotype differs from the shrinker phenotype of worms that have lost all GABAergic synaptic function [18], [35]. suggesting that GABAergic synaptic transmission is only partially affected in *ju89* mutants or that alterations in the synaptic transmission of cholinergic motor neurons or interneurons may also contribute to the locomotion phenotype. We found that *ju89* heterozygotes (*ju89/+)* moved in a pattern intermediate between wild-type and homozygous mutant worms and also exhibited weak variable defects in SNB-1::GFP expression, including irregular spaced puncta and a reduction in the number of dorsal puncta that varied between 10 and 20% (Table 1). The *tba-1(ok1135)* deletion removes almost 900 bp (70%) of the *tba-1* coding sequence and is a likely null allele (Figure 5B), *ok1135* animals have wild-type locomotion and SNB-1-GFP expression patterns (Table 1).

To determine if this weak dominant effect of *ju89* was due to gene dosage, we examined the SNB-1::GFP expression in worms that were heterozyzgous for a deficiency chromosome, (*qDf9*) or heterozygous for the *ok1135* deletion. The number of synapses based on SNB-1::GFP in *qDf9/+* heterozygotes and *ok1135*/+ heterozygotes was the same as in wild-type worms (Table 1), indicating that *tba-1* is not haploinsufficient. Moreover, the SNB-1::GFP defects of either *ju89/qDf9* heterozygotes or *ju89/ok1135* heterozygotes were less severe than *ju89/ju89* homozygotes, revealing that *ju89* behaves genetically as an altered function mutation. As further evidence that the *ju89* allele of *tba-1* can dominantly disrupt synapse and axon development, we amplified *tba-1* genomic DNA from wild-type and mutant animals, and injected it in parallel into wild-type worms (see Experimental Methods). Worms from fourteen of twenty-seven extragenic lines derived from *tba-1* DNA amplified from *ju89* mutants (*Extba-1R414)* exhibited SNB-1::GFP defects and uncoordinated movement similar to homozygous *ju89* mutants (Table 1, Figure 6B & D). We quantified the reduction in synapses for one *ExMtba-1* line and determined that the number of dorsal SNB-1::GFP puncta was reduced by an average of 35%. In comparison, all lines that expressed arrays of wild-type *tba-1* DNA injected at the same concentration were wild-type (Table 1; Figure 6A & C). Thus, the presence of the *ju89 tba-1* allele was sufficient to alter motor neuron development and function.

### Genetic interactions with loss-of-function *tbb-1* and *tbb-2* tubulin mutants

*tba-1*is one of two *C. elegans a*lpha-tubulins and two beta-tubulins expressed maternally during early embryogenesis [31]. RNAi knockdown of pairwise combinations of *tba-1, tba-2, tbb-1* and *tbb-2* and analysis of deletion alleles demonstrated that each set of tubulins have overlapping functions for embryonic viability [36]–[37]. However, these tubulins are not completely redundant in the embryo. The microtubule severing protein MEI-1 (katanin) acts preferentially on microtubules containing alpha-tubulin *tba-2* and the beta-tubulin *tbb-2* [38]–[39]. Gene-specific RNAi targeting of *tbb-2* also results in defects in centrosome positioning and ability of microtubules to interact properly with the cell cortex in early embryos that were not observed in single gene RNAi knockdown of the other three embryonic tubulins [26]. In later larval and adult stages, the expression pattern of these tubulins is more varied. *tba-1* and *tbb-2* are widely expressed throughout the *C. elegans* nervous system [31], [38], whereas neuronal expression of *tba-2* is restricted to a subset of cholinergic motor neurons (DB and VB neurons), sensory neurons and interneurons [40].

When we examined deletion alleles of *tba-1, tbb-1 and tbb-2* generated by the *C. elegans* Gene Knock-out Consortium for viability and defects in D neuron development we found similar results. We were unable to recover viable double mutant combinations of any of these three genes (*tba-2* deletion alleles were not available to include in this analysis). By contrast, 72% (169/235) of *tba-1(ju89); tbb-2(gk130)* and 68% (204/301) of *tba-1(ju89);tbb-1(gk207)* animals were viable at 20°C and were evaluated for expression of the SNB-1::GFP marker and DD morphology (Tables 1 & 2). This result supports that TBA-1-G414R subunits produced in *ju89* mutants can be incorporated into microtubules and retain essential functions in embryos.

Our analysis revealed that L1 larvae deficient for TBB-2 or TBB-1 exhibited DD axon outgrowth defects similar to, but weaker than, *tba-1(ju89)* mutants (Table 2):17% of *tbb-2*(*gk130)* axons were defective compared to 13.6% of defective axons in *tbb-1(gk207)* animals and 20% of axons that terminated prematurely in *tba-1(ju89)* mutants (Table 2). The frequency of axon defects was similar in the *tbb-2(gk130)* single and the *tba-1(ju89);tbb-2(gk130)* double mutant. The lack of a strong additive axon phenotype in the *tba-1(ju89);tbb-2(gk130)* double mutants suggested that axon outgrowth defects in *tba-1(ju89)* may involve loss of TBB-2 function. Indeed, when TBB-1 function was removed in the *tba-1(ju89); tbb-1(gk207)* double mutant, increased synthetic embryonic lethality was observed at 20°C, and DD motor neuron development was more severely affected in the surviving L1 larvae. 32% did not extend completely along the dorsal nerve cord (Tables 1 & 2), and DD neurons were mispositioned or missing in 10% (5/50) of the *tba-1(ju89);tbb-1(gk207)* double mutant animals (Figure 3E). Unlike the more complex phenotype of the dominant *ju89* single mutants, the severe loss of synapses in the *ju89; tbb-1(gk207)* double mutant and the decrease in synapses in *tbb-2(gk130)* loss-of-function animals (Table 1) appears to be caused by defective axon outgrowth.

Our genetic analysis indicates that the function of microtubules containing TBA-1-TBB-2 heterodimers is compromised in *ju89* mutants. DD neuron development may be less severely impaired in *ju89* single mutants because microtubules containing TBA-1/TBB1 heterodimers are still able to compensate for most alterations in TBA-1-TBB-2 heterodimer function during axon and synapse growth, or TBB-1 may form functional microtubules with another partially redundant alpha-tubulin expressed by the DD neurons. Interestingly, although the axon defects of *tbb-2(gk130)* single mutants were similar to *ju89* mutants (Table 2), most *gk130* mutants were not severely uncoordinated. The locomotion of *tbb-1(gk207)* single mutants was also similar to wild-type (data not shown). The total number of axon defects in *tba-1(ju89*); tbb*-2(gk207)* double mutants was also slightly improved compared to *ju89* single mutants (Table 2). These observations are consistent with our model that locomotion and synapse phenotypes in *ju89* mutants are partly independent of function during DD axon outgrowth.

## Discussion

Over the past decade, many structural components of the presynaptic active zone have been identified by biochemical or genetic strategies. One unresolved question in axon and presynaptic terminal differentiation is how the different components of the presynaptic specialization are regulated and transported to synaptic sites. Dynamic formation of nascent presynaptic structures occurs during axon outgrowth prior to synapse formation independent of contact with post-synaptic partners and is an integral step in establishing the connectivity pattern of a neuron [41]. Transport packets containing preassembled components of the active zone cytomatrix have also been detected in differentiating axons, and these transport packets localize independently of neurotransmitter vesicles [42]–[44]. Extension of the axon and transport of synaptic components are both dependent on the intricate regulation of microtubule dynamics and interactions with retrograde and anterograde motor proteins.

Maintenance of existing synapses and growth of new synapses are thought to rely on similar transport mechanisms. Between the late hatching and young adult stage, *C. elegans* goes through four larval molts and increases in size four-fold. As the animal grows, new membrane is added to existing motor neuron axons, and the neurons must either add new synapses or increase the size of existing synapses to maintain effective synaptic transmission. This transition has been documented in detail for the *C. elegans* DD neurons by Yeh and Zhen [27]. Using GFP markers and electron microscopy, they determined that the number of new GABAergic synapses increases by less than 30%, while each existing synapse increases in size by at least threefold. The distance between synapses also increases as new membrane is added to the axon.

Our analysis of *tba-1(ju89)* defects suggests that defects in microtubule structure or dynamics in the mutants compromises motor neuron axon and synapse growth. Our data indicates that the *tba-1(ju89)* mutation perturbs initial axon outgrowth, and that the mutants exhibit additional synapse defects. Many of the synaptic phenotypes we have documented in *ju89* mutants are consistent with defects in the biogenesis or trafficking of vesicles containing structural components of motor neuron synapses and axons, rather than a defect solely in synaptic vesicle transport. In Drosophila *imac* mutants, for example, synaptic boutons completely disappear from motor neurons due to loss of a kinesin-3 motor that is implicated in transport of active zone cytomatrix proteins as well as synaptic vesicles [45]. When *imac* function is removed, the distal tips of the axons also remain thin and undifferentiated, and synaptic vesicles are mislocalized. We identified quite similar, but weaker, phenotypes in the *tba-1(ju89)* mutants. Both the synaptic vesicle marker, SNB-1:GFP and the active zone marker, UNC-10::GFP, have reduced or absent expression at the distal tips of the D neurons, even when a thin neurite is still visible by epi-fluorescence microscopy. Indeed, the expression pattern of the synaptic vesicle marker SNB-1::GFP and active zone marker UNC-10::GFP in the GABAergic DD motor neuron were identical in *ju89* mutants. A small decrease in size of active zones and the vesicle pool detectable at these synapses was also observed in the cholinergic motor neurons by EM, while the proportion of synaptic vesicles/size of active zone was similar in wild-type and mutant animals. All of these data are consistent with a failure of the axons and synapses to grow properly as animals mature. The aberrant branching that increases with the age of the mutants may also reflect defects in microtubule stability and transport that slowly become more severe as axons grow to their full adult size.

Our genetic analysis demonstrates that the *ju89* mutation dominantly alters t*ba-1* function. Heterozygous *ju89* animals display locomotion and SNB-1-GFP phenotypes intermediate between wild-type and *ju89* homozygotes. We were also able to induce the mutant defects by expressing mutant TBA-1 protein in wild-type animals. By contrast, the loss-of-function *tba-1* allele appears nearly wild-type, likely due to overlapping function with other alpha-tubulins. Similar results have been observed during early cell divisions, where altered function alleles of tubulin mutations often have a more severe impact on microtubule function than null alleles [39], [46]. The genetic data that *ju89; tbb-2* and *ju89; tbb-1* double mutants are viable, while loss-of-function *tba-1 allele*, o*k1135* is lethal in combination with *tbb-1* or *tbb-2* null alleles, argues that the mutant TBA-1 protein is incorporated into microtubules and able to perform part of its wild-type functions. The absence of zygotic TBA-2 expression by the GABAergic D motor neurons and anterior cholinergic motor neurons [40] may explain why the *tba-1(ju89)* allele has a more deleterious affect on these neurons. Our genetic analysis indicates that TBA-1(R414) interacts with both TBB-1 and TBB-2 during neuronal development, but that MTs formed by TBA-1 and TBB-2 may be more severely affected for some functions. This result is consistent with another dominant allele of TBA-1, *or346*, that disrupts mitotic spindle positioning and causes severe embryonic lethality [37]. However, the relative contribution of each beta-tubulin to the *ju89* phenotypes may be more complex. Autoregulation and feedback between tubulins has been observed in *C. elegans* embryos, and could be a factor in later stages of development [46].

The *ju89* mutation is located near the beginning of helix 12 of the TBA-1 C-terminus near the alpha-beta tubulin heterodimer boundary, but it is not part of the intradimer interface. Cryo-EM studies indicate that H11 and H12 project to the outside of the microtubule and contribute to ridge and groove structures on the external face of microtubules, providing a binding surface for structural MAPs and motor proteins [47] The *ju89* mutation occurs in a critical location on the microtubule surface that could disrupt specific microtubule-associated proteins (MAPs). Motor proteins and other MAPs can selectively bind either the alpha or beta subunits of the microtubule polymer, and structural MAPs that stabilize protofilaments into the typical13-protofilament microtubule can potentially interact with both classes of tubulin. Some of the proteins identified to bind the alpha-tubulin subunit include the ebp family of microtubule plus-end binding proteins [15], tubulin destabilizing proteins op50/stathmin [48] and spastin [10], tubulin folding cofactor E [7], [49], doublecortin [50], [51] and the kinesin-3 motor Kif1A [52]. Because the genetic analysis suggests MTs containing TBA-1 and TBB2 may be especially impaired in *ju89* mutants, MAPs that specifically interact with TBB-2 could also be affected.

### Models for how *tba-1(ju89)* may affect microtubule dynamics and axon transport

*tba-1(ju89)* mutants exhibit pleiotropic defects in synapse and axon development that suggest TBA-1 interactions with several motors and structural MAPS may be altered that could alter microtubule dynamics or function. One likely candidate is the *C. elegans* homologue of Kif1A and IMAC: UNC-104. Similar to the phenotypes observed in Drosophila IMAC mutants, transport of synaptic vesicles is severely affected by loss-of-function mutations in *unc-104* [53]. The decrease in synaptic vesicles at *ju89* motor neuron synapses is consistent with partial loss of UNC-104-mediated transport. However, UNC-104 does not solely control localization of synaptic components such as UNC-10/RIM, UNC-13 and ELKS in *C. elegans* [26], and additional motors involved in transport of these proteins or other active zone proteins could also be perturbed in the *tba-1* mutants.

A second possibility is that the kinetics of tubulin folding and heterodimer formation is compromised by the *ju89* mutation. Mutations in tubulin-specific chaperonin E (TBCE), a key co-factor in tubulin dimer assembly and disassembly, have been shown to cause motor neuron axon defects and degeneration in mice [7]. The loss of distal microtubules and retrograde dying back of motor neuron axons in these mutants was subsequently shown to involve loss of TBCE function at the golgi apparatus where it regulates routing of axonal tubulin [49]. In a recent investigation of TBCE function in *Drosophila*, RNAi knockdown of either presynaptic or postsynaptic TBCE disrupted the distinctive microtubule loop structures present at the fly neuromuscular junction [54].

A third possibility is that *ju89* disrupts TBA-1 interactions with a specific MAP, such as doublecortin, that can stabilize MTS and MT interactions with motor proteins. Mutations in human and mouse alpha-tubulin, TUBA1A (TUB3A) cause lissencephaly [16]. Both the human lissencephaly R402H TUBA1A mutation and *tba-1(ju89)* G414R mutations are predicted to alter the H11–H12 loop of alpha-tubulin. Lissencephaly results from the failure of vertebrate cortical neurons to migrate properly during development, and is frequently caused by mutations in doublecortin (DCX) [55]. DCX is a potent microtubule stabilizer expressed by post-mitotic neurons that binds between the protofilaments of the MT polymer, promoting polymerization and stable microtubules that can support kinesin and dynein activity [54]–[55]. Analysis of the binding of DCX to microtubules indicates that the H11–H12 loop of alpha-tubulin can form an interface with DCX or the closely related dclk(doublecortin related kinase) [55]. Vertebrate DCX and Dclk are also present at axon termini in developing neurons [56]–[57], and defects in axon extension and transport occur in mice heterozygous for mutations in the Mouse homologues of these proteins [58]–[59]. Axon outgrowth defects and disorganized axon tracts were also identified recently in studies of stillborn human TUBA1A embryos [60] and in a broad spectrum of developmental brain disorders newly characterized in mammalian beta-tubulin mutants [61]. It is noteworthy that dominant mutations in the mammalian beta-tubulin TUBB3 result both in reduced microtubule dynamics and perturbed MT binding to the kinesin Kif21A in in vitro experiments, and are associated with defects in axon maintenance in addition to an earlier developmental affect on axon outgrowth [61].

The *C. elegans* genome contains a single doublecortin family member, the dclk protein ZYG-8. *zyg-8* is a maternal effect lethal gene in *C. elegans*; embryos from homozygous mutant hermaphrodites arrest due to defects in positioning the mitotic spindle [62]. It remains to be determined if TBA-1 interactions with TBCE, ZYG-8 or specific kinesins are altered in *ju89* mutants; these questions can be addressed by direct biochemical experiments and genetic modifier screens in the future. The similarity between the *tba-1(ju89)* phenotypes and neurodevelopmental defects reported in mammalian motor neuron diseases make the *C. elegans tba-1(ju89)* mutant a useful genetic tool to study the microtubule-mediated functions altered in these disorders and identify mechanisms that can stabilize axons and reverse synapse loss.

## Materials and Methods

### *C. elegans* strains and genetics

All strains were cultured at 20°C as described by Brenner [63]. *tba-1(ju89)* was isolated from an ethylmethane sulfonate (EMS) mutagenesis screen of N2 animals carrying the integrated chromosomal array *ju1s1 {P_ unc-25_SNB::GFP; lin-15*(+)*}.* The *ju89* mutation was backcrossed multiple times. Linkage mapping placed *ju89* to chromosome one based on the uncoordinated and syd (synapse defective) phenotype. Standard two and three factor mapping was used to refine a map position between *egl-33* and *lin-11*: *dpy-5* (9/10*) ju89* (0/7) *unc-29; dpy-5 (*20/22*) ju89 mom-5; unc-13 (*6/16) *ju89* (1/9) *egl-33; unc-29*(14/37) *ju89 lin-11; unc29hp6* (7/27) *ju89 dpy-24*.

Strains used in the study include RB1185: *tba-1(ok1135)I*; CZ2569: *tba-1(ju89)*I; JK1553: *ces-1qDf9/unc-29(e1072) lin-11(n566*)I; CB2167: *dpy-5(e61)*I *unc-13(e1091* I; EU459 *mom-5(zu193)unc-13(*e1091)/*hT2* I; *+/hT2* lll; *him-8(ec56)* IV; MT151: *egl-33(n151)*; SP1726: *unc-29(h1) hp6 dpy-24(s71)*I; VC364: *tbb-1(gk207*)III; VC167: *tbb-2(gk130)*III. The genotypes of other markers used are: *oxIs22 {UNC-49B::GFP}*; *juIs137 {*P*_flp-13_*SNB-1::GFP}; *juIs76 {*P*_unc-25_*GFP}; *juIs145 {*P*_flp-13_*GFP}; *hpIs61* {P*_unc-25_*UNC10-GFP; *juEx1368* {P*_unc-25_*mCherry::RAB-3} [64]. Strains carrying extragenic *tba-1* arrays were generated as described below.

#### Molecular biology

Cosmids and fosmids were provided by the Sanger Center, Hinxton, UK. A 10.5 kb PstI and PvuII fragment of genomic DNA derived from cosmid F26E4 was cloned into pBluescript to generate pCZ483. pCZ483 includes both genes of the *tba-1* operon (*tba-1* and *drsh-1*) and 2 kb of upstream genomic sequence. All other genomic subclones were deletion derivatives of pCZ483. pCZ485 was generated by digesting pCZ483 with BstZi71 and religating, resulting in a subclone in which exon 3 of *tba-1* was deleted. pCZ486 contains a deletion only in the *drsh-1* coding region and was constructed by digesting pCZ483 with SphI and religating. pCZ484 is a pBluescript subclone containing a PVUII- AccIII fragment of pCZ483 that encodes only *tba-1*(F26E4.8).

The *ju89* lesion was identified by determining the genomic sequence of all exons and introns of *tba-1* and 1.2 kb of sequence upstream of the 5′ end of *tba-1*. Genomic DNA from *ju89* mutants was generated by standard PCR techniques, and PCR products from three independent PCR reactions for each primer pair was used to obtain sequence from both strands (UC-Berkeley Sequencing Center). The following primer pairs were used to confirm *tbb-1* and *tbb-2* deletion alleles: *tbb-1*: 5′-CATTGATATTACCGGCTCGAGAC-3′ and 5′GTAGACATCGATTCTCTCCAGCT3′; *tbb-2(gk130)*: 5′TAGAAAGGTACTTGCGCTGA-3′ and 5′-GACAAGCTCAGCTCCTTCTGTG-3′.

#### Transgenic strains

Germline transformation was performed following standard *C. elegans* procedures [65]. Cosmid and fosmid DNA and subclones were injected at .5–2 ng/µl along with either 50 ng/µl of *pRF4* or 20 ng/µl P***_ttx–3_***GFP as selectable markers. The Expand Long PCR system (Roche) was used to amplify the 4.5 kb *tba-1* genomic region from N2 and *ju89* worms. Strains expressing either the PCR product amplified from wild-type DNA {*Extba-1*} or from the mutant *ju89* DNA {*Extba-1R414*} were constructed by injecting wild-type worms with 1 ng/ul of PCR product together with 20 ng/µl P***_ttx–3_***GFP as a selectable marker [66].

#### GFP and immunocytochemical analysis

Live GFP observation and imaging was performed with an HQ-FITC filter setup (Chroma, VT, USA) and 63X objective on a Zeiss AxioplanII with a Zeiss Axiocam imaging system and a Zeiss Pascal Confocal Microscope. Additional images were recorded using HQ-FITC and HQ-rhodamine filters and 60X objective on a Nikon E800 microscope equipped with a Coolsnap ES camera and Metamorph software. For imaging, adult animals were anesthetized using phenoxy propanol or pretreated in 2% parafomaldehyde for 20 minutes. L1 larvae were imaged without anesthetic. Whole-mount staining for anti-SNT-1 used a modification of paraformaldehyde fixation [67]. For anti-UNC-10 (Rim) staining, worms were prepared using Bouin's fixative [68]. Rabbit anti-SNT-1 and anti-UNC-10 antibodies were provided by Mike Nonet. Secondary mouse anti-rabbit antibodies were obtained from Jackson Laboratories.

### Analysis of axon outgrowth

The six dorsal D (DD) motor neurons were visualized with P*_flp-13_*GFP *(juIs145)*; P*_flp-13_*SNB-1::GFP *(juIs137)*; and P*_unc-25_*GFP (*juIs76)* transgenes. All 6 DD axons were evaluated in L1 larvae expressing *juIs76*, and DD1-DD5 axons were examined in L1s and adult worms that expressed the *juIs145* or *juIs137* reporter transgenes. To quantify DD axon outgrowth defects along the dorsal nerve cord, images were collected using a Coolsnap ES CCD camera (Roper) and Metamorph software. Metamorph or ImageJ software was used to measure the total dorsal nerve cord length and any detectable break or gap(s) present in the dorsal nerve cord for each animal. To assess changes in DD morphology between L1 and adult stages, L1s with no defects in *juIs145* GFP expression were selected, grown for 2 days at 20–22°C, and were then examined a second time by epi-fluorescence microscopy.

#### EM analysis

Young adult *juIs1 (wild-type)* and *tba-1(ju89); juIs1* animals were fixed in parallel using glutaraldehyde and osmium as described previously [21]. Serial thin sections (45 nm) were collected from the anterior of each animal, between the nerve ring and the vulva. A JEOL-1200 electron microscope equipped with a GATAN digital camera was used to photograph the ventral and dorsal nerve cords. GABAergic NMJs were identified as synapses made by the DD or VD neurons onto muscle arms. Cholinergic NMJs were identified as dyadic synapses onto both muscles and DD or VD neurons. The identity of the nerve processes in the nerve cords was determined by comparison to EM images from the *C. elegans* database [17]. Data was collected from one *juIs1* animals (1,500 sections) and two *ju89; juIs1* animals (400 sections each).

## References

[1] Roos J, Hummel T, Ng N, Klambt C, Davis GW (2000). *Drosophila* Futsch regulates synaptic microtubule organization and is necessary for synaptic growth.. Neuron.

[2] Hummel T, Krukkert K, Roos J, Davis G, Klambt C (2000). *Drosophila* Futsch/22C10 is a MAP1B-like protein required for dendritic and axonal development.. Neuron.

[3] Eaton BA, Fetter RD, Davis GW (2002). Dynactin is necessary for synapse stabilization.. Neuron.

[4] Ruiz-Canada C, Ashley J, Moeckel-Cole S, Drier E, Yin J (2004). New synaptic bouton formation is disrupted by misregulation of microtubule stability in aPKC mutants.. Neuron.

[5] Desai A, Mitchison TJ (1997). Microtubule polymerization dynamics.. Ann Rev Cell & Dev Biol.

[6] Szymanski D (2002). Tubulin folding cofactors: half a dozen for a dimer.. Curr Biol.

[7] Bommel H, Xie G, Rossoll W, Wiese S, Jablonka S (2002). Missense mutation in the tubulin-specific chaperone E (Tbce) gene in the mouse mutant progressive motor neuronopathy, a model of human motoneuron disease.. J Cell Biol.

[8] LaMonte BH, Wallace KE, Holloway BA, Shelly SS, Ascano J (2002). Disruption of dynein/dynactin inhibits axonal transport in motor neurons causing late-onset progressive degeneration.. Neuron.

[9] Puls I, Jonnakuty C, LaMonte BH, Holzbaur E, Tokita M (2003). Mutant dynactin in motor neuron disease.. Nat Genet.

[10] Errico A, Ballabio A, Rugarli E (2002). Spastin, the protein mutated in autosomal dominant hereditary spastic paraplegia, is involved in microtubule dynamics.. Human Mol Genetics.

[11] Moccia R, Chen D, Lyles V, Kapuya E, Kalachikov S (2003). An unbiased cDNA library prepared from isolated Aplysia sensory neuron processes is enriched for cytoskeletal and translational mRNAs.. J Neurosci.

[12] Imai Y, Takahashi R (2004). How do Parkin mutations result in neurodegeneration?. Curr Opin Neurobiol.

[13] Yang F, Jiang Q, Zhao J, Ren Y, Sutton MD (2005). Parkin stabilizes microtubules through strong binding mediated by three independent domains.. J Biol Chem.

[14] Asakawa K, Kume K, Kanai M, Goshima T, Miyahara K (2006). The V260I mutation in fission yeast alpha-tubulin Atb2 affects microtubule dynamics and EB1-Mal3 localization and activates the Bub1 branch of the spindle checkpoint at the nuclear envelope.. Mol Biol Cell.

[15] Levy JR, Sumner CJ, Caviston JP, Tokito MK, Ranganathan S (2006). A motor neuron disease-associated mutation in p150Glued perturbs dynactin function and induces protein aggregation.. J Cell Biol.

[16] Keays DA, Tian G, Poirer K, Huang G-J, Siebold C (2007). Mutations in alpha-tubulin cause abnormal neuronal migration in mice and lissencephaly in humans.. Cell.

[17] White JG, Southgate J, Thompson N, Brenner S (1986). The structure of the nervous system of the nematode *Caenorhabditis elegans*.. Phil Trans Royal Soc.

[18] Jin Y, Jorgensen E, Hartwieg E, Horvitz HR (1999). The *Caenorhabditis elegans* gene *unc-25* encodes glutamic acid decarboxylase and is required for synaptic transmission but not synaptic development.. J Neurosci.

[19] Nonet ML (1999). Visualization of synaptic specializations in live *C. elegans* with synaptic vesicle protein-GFP fusions.. J Neurosci Methods.

[20] Hallam SJ, Goncharov A, McEwen J, Baran R, Jin Y (2002). SYD-1, a presynaptic protein with PDZ, C2 and rhoGAP-like domains, specifies axon identity in *C. elegans*.. Nat Neurosci.

[21] Zhen M, Jin Y (1999). The liprin protein SYD-2 regulates the differentiation of presynaptic termini in *C. elegans*.. Nature.

[22] Zhen M, Huang X, Bamber B, Jin Y (2000). Regulation of presynaptic terminal organization by *C. elegans* RPM-1, a putative guanine nucleotide exchanger with a RING-H2 finger domain.. Neuron.

[23] Crump JG, Zhen M, Jin Y, Bargmann CI (2001). The SAD-1 kinase regulates presynaptic vesicle clustering and axon termination.. Neuron.

[24] Byrd DT, Kawasaki M, Walcoff M, Hisamoto N, Matsumoto K (2001). UNC-16, a JNK-signaling scaffold protein, regulates vesicle transport in *C. elegans*.. Neuron.

[25] Bamber BA, Beg AA, Twyman RE, Jorgensen EM (1999). The *Caenorhabditis elegans unc-49* locus encodes multiple subunits of a heteromultimeric GABA receptor.. J Neurosci.

[26] Deken SL, Vincent R, Hadwiger G, Liu Q, Wang WZ (2005). Redundant localization mechanisms of RIM and ELKS in *Caenorhabditis elegans*.. J Neurosci.

[27] Yeh E, Kawano T, Weimer RM, Bessareau J-L, Zhen M (2005). Identification of genes involved in synaptogenesis of *Caenorhabditis elegans*.. J Neurosci.

[28] Li C, Kim K, Nelson LS (1999). FMRFamide-related neuropeptide gene family in *Caenorhabditis elegans*.. Brain Res.

[29] Sakaguchi-Nakashima A, Meir JY, Jin Y, Matsumoto K, Hisamoto N (2007). LRK-1, a *C. elegans* PARK8-related kinase, regulates axonal-dendritic polarity of SV proteins.. Curr Biol.

[30] elegans Sequencing Consortium C (1998). Genome sequence of the nematode *C. elegans*: A platform for investigating biology.. Science.

[31] Baugh LR, Hill AA, Slonim DK, Brown EL, Hunter C (2003). Composition and dynamics of the *Caenorhabditis elegans* early embryonic transcriptome.. Development.

[32] Fukushige T, Yasuda H, Siddiqui SS (1995). Selective expression of the *tba-1* alpha tubulin gene in a set of mechanosensory and motor neurons during the development of *Caenorhabditis elegans*.. Biochimica et Biophysica Acta - Gene Structure and Expression.

[33] Nogales E, Wolf SG, Downing KH (1998). Structure of the αβ-tubulin dimer by electron crystallography.. Nature.

[34] Lowe J, Li H, Downing KH, Nogales E (2001). Refined structure of alpha beta-tubulin at 35 A resolution.. J Mol Biol.

[35] McIntire Sl, Jorgensen EM, Kaplan J, Horvitz HR (1993). The GABAergic nervous system of *Caenorhabditis elegans.*. Nature.

[36] Wright AJ, Hunter C (2003). Mutations in a beta-tubulin disrupt spindle orientation and microtubule dynamics in the early *Caenorhabditis elegans* embryo.. Mol Biol Cell.

[37] Phillips JB, Lyczak R, Ellis GC, Bowerman B (2004). Roles for two partially redundant alpha-tubulins during mitosis in early *Caenorhabditis elegans* embryos.. Cell Motil Cytoskeleton.

[38] Lu C, Srayko M, Mains PE (2004). The *Caenorhabditis elegans* Microtubule-severing complex MEI-1/MEI-2 katanin interacts differently with two superficially redundant beta-tubulin isotypes.. Mol Biol Cell.

[39] Lu C, Mains PE (2005). Mutations of a redundant alpha-tubulin gene affect *C. elegans* early embryonic cleavage via MEI-1/katanin dependent and independent pathways.. Genetics.

[40] Fukushige T, Yasuda H, Siddiqui SS (1993). Molecular cloning and developmental expression of the alpha-2 tubulin gene of Caenorhabditis elegans.. J Mol Biol.

[41] Jin Y, Garner C (2008). Molecular mechanisms of synapse differentiation.. Ann Rev Cell Dev Biol.

[42] Ahmari SE, Buchanan J, Smith SJ (2000). Assembly of presynaptic active zones from cytoplasmic transport packets.. Nat Neurosci.

[43] Zhai RG, Vardinon-Friedman H, Cases-Langhoff C, Becker B, Gundelfinger ED (2001). Assembling the presynaptic active zone: a characterization of an active zone precursor vesicle.. Neuron.

[44] Shapira M, Zhai RG, Dresbach T, Bresler T, Torres VI (2003). Unitary assembly of presynaptic active zones from Piccolo-Bassoon transport vesicles.. Neuron.

[45] Pack-Chung E, Kurshan TP, Dickman KD, Schwarz LT (2007). A *Drosophila* kinesin required for synaptic bouton formation and synaptic vesicle transport.. Nat Neurosci.

[46] Ellis GC, Phillips JB, O'Rourke S, Lyczak, R, Bowerman, B (2004). Maternally expressed and partially redundant beta-tubulins in *Caenorhabditis elegans* are autoregulated.. J Cell Sci.

[47] Al-Bassam J, Ozer RS, Safer D, Halpain S, Milligan, RA (2002). MAP2 and tau bind longitudinally along the outer ridges of microtubule protofilaments.. J Cell.

[48] Larsson N, Marklund U, Gradin HM, Bratts G, Gullberg M (1997). Control of microtubule dynamics by oncoprotein 18: dissection of the regulatory role of multisite phosphorylation during mitosis.. Mol Cell Biol.

[49] Schaefer MK, Schmalbruch H, Buhler E, Lopez C, Marin N (2007). Progressive motor neuronopathy: a critical role of the tubulin chaperone TBCE in axonal tubulin routing from the golgi apparatus.. J Neurosci.

[50] Moores CA, Perderiset M, Francis F, Chelly J, Houdusse A (2004). Mechanism of microtubule stabilization by doublecortin.. Mol Cell.

[51] Moores CA, Perderiset M, Kappeler C, Kain S, Drummond D (2006). Distinct roles of doublecortin modulating the microtubule cytoskeleton.. EMBO J.

[52] Kikkawa M, Hirokawa N (2006). High-resolution cryo-EM maps show the nucleotide binding pocket of KIF1A in open and closed conformations.. EMBO J.

[53] Hall DH, Hedgecock EM (1991). Kinesin-related gene unc-104 is required for axonal transport of synaptic vesicles in *C. elegans*.. Cell.

[54] Jin S, Pan L, Liu Z, Wang Q, Xu Z (2009). *Drosophila* tubulin-specific chaperone E functions at neuromuscular synapses and is required for microtubule network formation.. Development.

[55] Kerjan G, Gleeson JG (2007). Genetic mechanisms underlying abnormal neuronal migration in classical lissencephaly.. Trends Genet.

[56] Schaar BT, Kinoshita K, McConnell SK (2004). Doublecortin microtubule affinity is regulated by a balance of kinase and phosphatase activity at the leading edge of migrating neurons.. Neuron.

[57] Friocourt G, Koulakoff A, Chafey P, Boucher D, Fauchereau F (2003). Doublecortin functions at the extremities of growing neuronal processes.. Cereb Cortex.

[58] Deuel TA, Lui JS, Corbo JC, Yoo SY, Rorke-Adams LB (2006). Genetic interactions between doublecortin and doublecortin-like kinase in neuronal migration and axon outgrowth.. Neuron.

[59] Koizumi H, Tanaka T, Gleeson JG (2007). Doublecortin-like kinase functions with doublecortin to mediate fiber tract decussation and neuronal migration.. Neuron.

[60] Fallet-Bianco C, Loeuillet L, Poirier K, Loget P, Chapon F (2008). Neuropathological phenotype of a distinct form of lissencephaly associated with mutations in TUBA1A.. Brain.

[61] Tischfield MA, Baris HN, Wu C, Rudolph G, Maldergem Van (2010). Human TUBB3 mutations perturb microtubule dynamics, kinesin interactions and axon guidance.. Cell.

[62] Gönczy P, Bellanger JM, Kirkham M, Pozniakowski A, Baumer K (2001). *zyg-8*, a gene required for spindle positioning in *C. elegans*, encodes a doublecortin-related kinase that promotes microtubule assembly.. Dev Cell.

[63] Brenner S (1974). The genetics of *Caenorhabditis elegans*.. Genetics.

[64] Brown HM, Van Epps HA, Goncharov A, Grant BD, Jin Y (2009). The JIP3 scaffold protein UNC-16 regulates RAB-5 dependent membrane trafficking in *C. elegans*.. Dev Neurobiol.

[65] Mello C, Fire A (1995). DNA transformation.. Methods Cell Biol.

[66] Hobert O, Mori I, Yamashita Y, Honda H, Oshima YM (1997). Regulation of interneuron function in the *C. elegans* thermoregulatory pathway by the *ttx-3* LIM homeobox gene.. Neuron.

[67] Finney M, Ruvkun G (1990). The *unc-86* gene product couples cell lineage and cell identity in *C. elegans*.. Cell.

[68] Nonet ML, Staunton JE, Kilgard MP, Fergestad T, Hartwieg E (1997). *Caenorhabditis elegans rab-3* mutant synapses exhibit impaired function and are partially depleted of vesicles.. J Neurosci.

